# A Rare PKHD1 Frameshift Variant Identified in a Prenatally Diagnosed ARPKD Case From a Consanguineous Jordanian Family

**DOI:** 10.1155/crog/4656543

**Published:** 2026-09-12

**Authors:** Oqba Al-Kuran, Reham Albadaineh, Adnan Almallah, Dana Al-atrash, Duaa Dahbour, Lama AL-Mehaisen

**Affiliations:** ^1^ Department of Obstetrics and Gynecology, Jordan University Hospital, The University of Jordan, Amman, Jordan, ju.edu.jo; ^2^ School of Medicine, The University of Jordan, Amman, Jordan, ju.edu.jo; ^3^ Department of Obstetrics and Gynecology, Medical School, Al-Balqa Applied University, Al-salt, Jordan, bau.edu.jo

**Keywords:** ARPKD, exome sequencing, PKD, ultrasonography, variant consanguinity

## Abstract

**Introduction:**

Polycystic kidney disease (PKD) is a condition that encompasses the development of multiple benign cysts in the kidneys and other organs. Hereditary PKD can be classified as autosomal dominant polycystic kidney disease (ADPKD) or autosomal recessive polycystic disease (ARPKD). These two forms differ primarily in their age of onset and clinical presentation. ADPKD is usually diagnosed during young adulthood, whereas ARPKD presents a wide range of clinical manifestations with approximately one‐third of patients presenting before the first year of life, one‐third between 1 and 20 years of age, and the remaining one‐third after 20 years of age. ARPKD represents the severe form, typically presenting in infancy with significant renal and hepatic involvement. Hereditary and congenital forms are known to be caused by certain genetic defects associated with dysfunction of the primary cilia in the tubular epithelium. To the best of our knowledge, no similar cases have been reported in Jordan or neighboring countries.

**Case Presentation:**

We report a case of ARPKD in one fetus of a twin pregnancy. Prenatal ultrasonography at the 20th week of gestation revealed characteristic findings of ARPKD in one fetus. The twins were delivered at 36 weeks gestation. The affected twin died shortly after birth because of severe respiratory distress. Postnatal exome sequencing (ES) on the deceased infant identified a pathogenic homozygous variant in the polycystic kidney and hepatic disease (*PKHD1)* gene, supporting the genetic diagnosis of ARPKD.

**Discussion:**

Management of ARPKD requires multidisciplinary care, emphasizing supportive therapy and monitoring for complications. Genetic counseling is pivotal, highlighting the risk of disease transmission and the role of prenatal genetic screening for at‐risk families.

**Conclusions:**

Our case highlights the importance of early diagnosis, comprehensive management, and genetic counseling in ARPKD, particularly within the Jordanian population as well as neighboring demographics with high rates of consanguinity.

## 1. Introduction

Polycystic kidney disease (PKD) is a condition that encompasses the development of multiple benign cysts in the kidneys and other organs. Hereditary PKD can be classified as autosomal dominant polycystic kidney disease (ADPKD) or autosomal recessive polycystic disease (ARPKD) with the main difference between the two being the time of diagnosis; ADPKD is usually diagnosed during young adulthood [1], whereas ARPKD has a spectrum of presentations with approximately one‐third of patients presenting before 1 year of age, one‐third between 1 and 20 years of age, and one‐third after 20 years of age [2]. Hereditary and congenital forms are known to be caused by certain genetic abnormalities associated with dysfunction in the tubular epithelium′s primary cilia [3].

ARPKD, previously called infantile PKD, is characterized by cystic dilations of the renal collecting ducts and developmental defects of hepatobiliary ductal plate remodeling, which result in varying degrees of congenital hepatic fibrosis. The majority of cases are linked to variants in the polycystic kidney and hepatic Disease 1 (*PKHD1*) gene [4, 5]. The pattern of clinical presentations of ARPKD varies widely [6–10]. Patients who present during infancy are more likely to have severe kidney disease and poor survival rates, whereas patients diagnosed during adolescence or adulthood typically present with symptoms related to congenital hepatic fibrosis [11, 12].

Regarding diagnosis, molecular genetic testing is considered the gold standard and should be offered when it is available to identify the underlying variants [13]. Exome sequencing (ES) is highly effective at identifying different variants, is cost‐effective for detection, greatly reduces detection time, and provides accurate results [14]. If unavailable, the clinical diagnosis of ARPKD is typically made by an abdominal ultrasound [15]. Management of ARPKD is mainly supportive as there is no curative intervention. This may include the management of neonatal respiratory distress, arterial hypertension, end‐stage kidney disease (ESKD), portal hypertension, and recurrent cholangitis.

We describe a case of a female neonate with ARPKD diagnosed at 20 weeks of gestation by ultrasound. Postnatal ES in the deceased newborn revealed a rare homozygous pathogenic variant c.11408dup (p.Ala3804GlyfsTer9) in the *PKHD1* gene.

## 2. Materials and Methods

Written informed consent was obtained from both parents (the mother and the father of the proband) for participation in genetic testing, clinical evaluation, and for the publication of any potentially identifiable clinical information or images included in this report.

Genomic DNA was extracted from peripheral blood samples collected from the proband and parents. The WES for the proband was carried out using Agilent′s SureSelect Human All Exon V6 kit following manufacturer′s instructions and sequenced on an Illumina platform. Around 98.75% of genes were covered at least > 10x. The reads were aligned to GRCh37/hg19 genome assembly. All pathogenic variants reported in HGMD and in ClinVar, and all variants with minor allele frequency (MAF) < 1% in gnomAD database were considered. All variants, except nonsense and nonsynonymous variants, as well as insertions and deletions (indels), and splice site variants (+/−20 intronic bases), were excluded from the analysis. In silico prediction tools were also used to predict the functional impact of identified variants. All variants were classified according to the ACMG guidelines (Table 1) [16].

**Table 1:** Rules for combining criteria to classify sequence variants.

Classification	Criteria
Pathogenic	i. Very strong (PVS1) and
	a. ≥ 1 Strong (PS1–PS4) or
	b. ≥ 2 Moderate (PM1–PM6) or
	c. 1 Moderate (PM1–PM6) and 1 supporting (PP1–PP5) or
	d. ≥ 2 Supporting (PP1–PP5)
	ii. ≥ 2 Strong (PS1–PS4) or
	iii. Strong (PS1–PS4) and
	a. ≥ 3 Moderate (PM1–PM6) or
	b. 2 Moderate (PM1–PM6) and ≥ 2 supporting (PP1–PP5) or
	c. 1 Moderate (PM1–PM6) AND ≥ 4 supporting (PP1–PP5)

Likely pathogenic	i. 1 Very strong (PVS1) and 1 moderate (PM1–PM6) or
	ii. 1 Strong (PS1–PS4) and 1–2 moderate (PM1–PM6) or
	iii. 1 Strong (PS1–PS4) and ≥ 2 supporting (PP1–PP5) or
	iv. ≥ 3 Moderate (PM1–PM6) or
	v. 2 Moderate (PM1–PM6) and ≥ 2 supporting (PP1–PP5) or
	vi. 1 Moderate (PM1–PM6) and ≥ 4 supporting (PP1–PP5)

Benign	i. 1 Stand‐alone (BA1) or
	ii. ≥ 2 Strong (BS1–BS4)

Likely benign	i. 1 Strong (BS1–BS4) and 1 supporting (BP1–BP7) or
	ii. ≥ 2 Supporting (BP1–BP7)

Uncertain significance	i. Other criteria shown above are not met or
	ii. The criteria for benign and pathogenic are contradictory

## 3. Case Presentation

**Figure 1:**
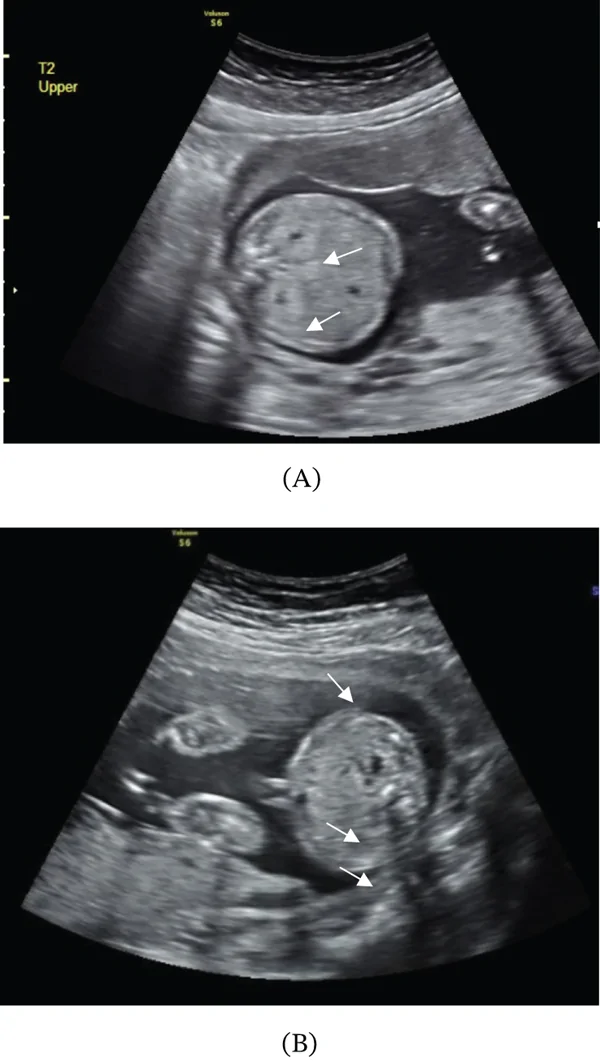
Ultrasound findings for (A) T2 and (B) T1. Both kidneys appearing brighter than cotwin suggesting polycystic kidney disease. Prenatal ultrasound findings at 20 weeks of gestation in a dichorionic diamniotic twin pregnancy. (A) Transverse ultrasound image of the affected twin (T2) demonstrating bilaterally enlarged, diffusely hyperechogenic kidneys (arrows), consistent with autosomal recessive polycystic kidney disease (ARPKD). Note the loss of corticomedullary differentiation and the relatively reduced amniotic fluid volume compared with the cotwin. (B) Transverse ultrasound image of the unaffected cotwin (T1) demonstrating normal renal echogenicity and architecture (arrows) with normal amniotic fluid volume, included for comparison.

A healthy 25‐year‐old Gravida 2, Para 1, her first pregnancy (P1) had PCKD and died immediately postpartum with no postmortem or any genetic testing. The patient presented to our clinic at 20 weeks of gestation. Ultrasound showed dichorionic diamniotic twins; measurement of the first twin (T1) was consistent with age, and fetal anatomy was normal with borderline right renal pelvis dilation (4.1 mm) and a normal amount of amniotic fluid. Measurements of the second twin (T2) were also compatible with age with apparently normal fetal anatomy other than both kidneys appearing brighter than the cotwin suggesting PKD [Figure 1]; the amniotic fluid volume was within normal limits but notably less than that of the cotwin.

The pregnancy was uneventful, and the mother did not have any significant symptoms. However, oligohydramnios was noted on subsequent visits, raising the risk of neonatal pulmonary hypoplasia after delivery. The mother had no chronic illnesses and did not smoke or consume alcohol during her pregnancy. There was no family history of PKD or similar conditions, though the parents were first cousins.

The twins were delivered at 36 weeks of gestation at the Al‐Basheer Hospital (not the University of Jordan Hospital) by cesarean section. The twins were referred to our unit, both females, and they were admitted to the NICU because of respiratory distress; T1 recovered after 7 days and was doing well, the infant was genetically tested and was normal, whereas T2 unfortunately died 1 day after delivery. Hence birth weights, APGAR scores, and respiratory support details were not available. Autopsy was not done as the patient was delivered in a community hospital setting and could not be referred to our tertiary hospital. Hence, the patient′s and her infants′ record could not be obtained.

Postnatal ES results of the deceased newborn (T2) were received approximately 1 month after the infant′s death, as ES typically requires 3–4 weeks for results. ES homozygous pathogenic variant (c.11408dup) was identified in the *PKHD1* gene. The identified duplication variant (c.11408dup) lies in Exon 64 of the *PKHD1* gene and is predicted to cause a frameshift and consequent premature termination of the protein (p.Ala3804GlyfsTer9). This resultant protein will likely lack the transmembrane and C‐terminal cytoplasmic domains of the protein, which would likely result in loss‐of‐function. This variant is absent in public databases such as gnomAD, 1000 genome, and HGMD. A limitation of this report is that the identified variant was not confirmed by Sanger sequencing. However, parental carrier testing confirmed that both parents are heterozygous (healthy carriers), which confirms the autosomal recessive inheritance of the disease. Based on ACMG guidelines as shown in Table (16), this variant was classified as “pathogenic” (PVS1_Very Strong, PM3_Strong, and PM2_Supporting).

A multidisciplinary team is usually needed to manage ARPKD, but for a number of reasons, this was not possible in our situation. Early intervention chances were restricted because the mother did not get regular prenatal care. The window for specialized management was also shortened by the rapid fetal demise, and Jordan′s resource shortage made it even harder to assemble a full multidisciplinary team. Consequently, the primary emphasis of care was genetic diagnosis and urgent newborn support.

## 4. Discussion

ARPKD is an inherited disorder with most cases arising from variants in the *PKHD1* gene on Chromosome 6p21 which codes for fibrocystin, a large integral membrane protein [4, 5]. There are more than 750 reported variants in *PKHD1* [7]. The *DZIP1L* gene is a secondary locus for ARPKD, though less frequently than *PKHD1* [16]. The estimated incidence of ARPKD is 1 in 20,000 live births with a carrier frequency of 1/70 [3, 15, 17], with almost one third of patients presenting before 1 year of age [2].

Clinical presentation depends on the age and the extent of hepatic or renal involvement [2, 6–10]. Prenatal diagnosis is possible by ultrasonography after 24 weeks′ gestation. Though normal antenatal ultrasounds do not rule out ARPKD [18, 19].

Neonates may present with respiratory distress secondary to pulmonary hypoplasia [11, 15, 20, 21], impaired renal function that may progress to ESKD [21–26], and hepatobiliary manifestations including hepatomegaly, portal hypertension, and recurrent cholangitis [10, 27–30]. Feeding difficulties [31], thrombocytopenia [32], neurocognitive dysfunction [33], and left ventricular hypertrophy [34] may also be noted, as well as growth delay.

Early diagnosis and intervention are vital in ARPKD. Genetic testing is the preferred diagnostic tool to pinpoint the specific variants involved [35]. When genetic testing is not available for any reason, clinicians often rely on abdominal ultrasound to diagnose ARPKD, showing enlarged echogenic kidneys with inadequate corticomedullary differentiation and concurrent liver abnormalities [3]. Magnetic resonance imaging (MRI) may prove beneficial if still not confident about the diagnosis. Kidney biopsy is not a requisite for ARPKD diagnosis [31].

Following prenatal suspicion of ARPKD, serial ultrasonographic surveillance was performed to monitor fetal renal size, amniotic fluid volume, and fetal growth by the maternal–fetal medicine team and demonstrated progressive oligohydramnios, which indicated an increased risk of pulmonary hypoplasia.

As there is no available cure or disease‐modifying therapy, management is primarily supportive and focuses on trying to prevent and manage disease‐related complications [3]. The best care requires a multidisciplinary team, including specialists in maternal–fetal medicine (perinatology), neonatology, pediatric nephrology, hepatology, genetics, and other relevant disciplines [3]. Following a prenatal diagnosis, serial ultrasonographic assessment of fetal kidney size and amniotic fluid volume is recommended, with delivery planned at a tertiary center with neonatal intensive care facilities [3].

Our case reports the obstetric challenges of managing a dichorionic diamniotic twin pregnancy complicated by ARPKD affecting only one fetus. Whereas the affected twin demonstrated progressive renal disease and worsening oligohydramnios, the cotwin remained structurally normal with appropriate growth. Pregnancy management required balancing the risks of fetal deterioration in the affected twin against the risks of iatrogenic prematurity for the unaffected cotwin. This required individualized management with close fetal surveillance and multidisciplinary decision‐making regarding the timing and mode of delivery.

The affected fetus demonstrated progressive renal disease with worsening oligohydramnios; the cotwin remained structurally normal with proper fetal growth. Pregnancy management required understanding the risks of continuing the pregnancy for the affected fetus and the risks of prematurity for the unaffected cotwin. After birth, management should focus on respiratory stabilization, nutritional support, and close monitoring of blood pressure, renal function, electrolyte balance, and growth.

Infants who progress to ESKD may require dialysis, even though chronic dialysis during the first month of life can carry considerable morbidity and mortality [36]. Long‐term management includes controlling hypertension, promptly treating urinary tract infections, and monitoring for hepatobiliary complications such as portal hypertension and recurrent cholangitis [31]. For patients with ESKD, kidney transplantation is the preferred renal replacement therapy.

The prognosis of ARPKD is mainly determined by the severity of renal and hepatic involvement, with earlier clinical presentation generally reflecting more severe disease [17, 20, 21]. Neonates with advanced renal disease mainly associated with pulmonary hypoplasia have the poorest outcomes (reported mortality of 30%) [20, 21], patients who survive the neonatal period have an approximately 80% chance beyond 15 years of age [17, 20, 21, 37, 38].

Genetic counseling is an essential component of patient care. Parents should be informed that each subsequent pregnancy carries a 25% risk of an affected child and a 50% chance of having a carrier infant. In families with an identified pathogenic variant, prenatal genetic testing and preimplantation genetic testing are available options [39–43] and evaluation of asymptomatic siblings for congenital hepatic fibrosis should be considered [39–43].

Parents were counseled about the expected pregnancy progression, the likelihood of pulmonary hypoplasia, potential need for intensive neonatal resuscitation, dialysis, and long‐term renal replacement therapy in survivors. When a pathogenic familial variant has been identified, possible targeted prenatal diagnosis through chorionic villus sampling or amniocentesis, as well as preimplantation genetic testing, should be discussed in future pregnancies.

The variant c.11408dup (p.Ala3804GlyfsTer9) lies in Exon 64 of the 67‐exon *PKHD1* transcript, immediately upstream of the transmembrane and C‐terminal cytoplasmic domains of fibrocystin [6, 7]. The full‐length protein comprises a 3858‐amino acid extracellular domain, a single transmembrane domain, and a short C‐terminal cytoplasmic tail [6, 7], both of which are eliminated by the frameshift‐induced truncation at approximately residue 3812—consistent with predicted loss‐of‐function. Loss of the C‐terminal cytoplasmic domain is predicted to abolish this regulatory function, leading to increased SRC–STAT3 signaling, a pathway implicated in renal cytogenesis.

The severe neonatal outcome in our case, the prenatal findings of bilateral enlarged hyperechogenic kidneys followed by progressive oligohydramnios closely mirrored the severe molecular phenotype, further supporting the association between biallelic truncating *PKHD1* variants and severe perinatal disease. [32]. Nearby ClinVar‐documented truncating variants in the same distal region—p.Phe3813fs (RCV000670941.1) and p.Val3926fs (RCV000412407.2)—are classified as pathogenic/likely pathogenic for ARPKD, and their convergence with our identified variant in the same functionally critical region provides additional contextual support for its pathogenicity.

Our case report describes a unique finding in a Jordanian patient with ARPKD. The patient harbored a homozygous pathogenic variant (c.11408dup) in the *PKHD1* gene. To the best of our knowledge, this variant has not been previously reported in population databases; however, it has been previously reported in a patient with ARPKD who died in the neonatal period (PMID: 16133180).

The identification of this rare variant highlights the importance of integrating prenatal imaging with molecular diagnosis in pregnancies complicated by suspected ARPKD. Early recognition of the characteristic ultrasound findings allows early referral to a maternal–fetal medicine unit, multidisciplinary counseling, serial fetal surveillance, and delivery planning in tertiary centers with neonatal intensive care facilities, thereby optimizing perinatal and neonatal outcomes.

## 5. Limitation

Sanger sequencing was not done at that time.

## Author Contributions

Oqba Al‐Kuran: Conceptualization, project administration, and investigation. Reham Albadaineh: Data curation. Adnan Almallah: Resources and software. Dana Al‐atrash: Data curation and resources. Duaa Dahbour: Data curation and resources. Lama AL‐Mehaisen: Writing and review and editing.

## Funding

No funding was received for this manuscript.

## Disclosure

All authors have read and approved the final version of the manuscript. Professor Lama AL‐Mehaisen (corresponding author and manuscript guarantor) had full access to all of the data in this study and takes complete responsibility for the integrity of the data and the accuracy of the data analysis. Professor Lama AL‐Mehaisen (manuscript guarantor) affirms that this manuscript is an honest, accurate, and transparent account of the study being reported; that no important aspects of the study have been omitted; and that any discrepancies from the study as planned have been explained. No artificial intelligence (AI) tools were used in the preparation of this manuscript. All writing, analysis, and interpretation were performed by the authors.

## Ethics Statement

This case report did not require IRB approval as it does not constitute human subjects research under the applicable institutional guidelines (single case report with no experimental intervention). Written informed consent was obtained from both the mother and the father of the proband for participation in genetic testing and for the publication of any potentially identifiable images or data included in this article. The study was conducted in accordance with the Declaration of Helsinki.

## Consent

Written informed consent was obtained from the mother of the proband for the publication of any potentially identifiable images or data included in this article.

## Conflicts of Interest

None of the authors have a conflict of interest to disclose.

## Data Availability

Data are available on request from authors.
